# Supplementary material for: Ligand and G-protein selectivity in the κ-opioid receptor
Source: Nature. 2023 May 3;617(7960):417–25. doi: 10.1038/s41586-023-06030-7 (PMC10172140; doi:10.1038/s41586-023-06030-7)
Supplement: Supplementary file 1 — Supplementary Methods (allosteric IC50 shift model equation), Supplementary Figs. 1–6 and Supplementary Tables 1–17. [file 41586_2023_6030_MOESM1_ESM.docx]

**Ligand and G-protein selectivity in κ-opioid receptor**

Jianming Han^1,2^, Jingying Zhang^3,4,#^, Antonina L. Nazarova^5^, Sarah M. Bernhard^1,2^, Brian E. Krumm^6^, Lei Zhao^1^, Jordy Homing Lam^5^, Vipin A. Rangari^2^, Susruta Majumdar^1,2,9^, David E. Nichols^7^, Vsevolod Katritch^5^, Peng Yuan^3,4,#^, Jonathan F. Fay^8,^*, Tao Che^1,2,9,^*

^1^Department of Anesthesiology, Washington University in St. Louis, St. Louis, MO, USA.

^2^Center for Clinical Pharmacology, University of Health Sciences and Pharmacy in St. Louis and Washington University School of Medicine, St. Louis, MO, USA.

^3^Department of Cell Biology and Physiology, Washington University School of Medicine, St. Louis, MO, USA

^4^Center for the Investigation of Membrane Excitability Diseases, Washington University School of Medicine, St. Louis, MO, USA

^5^Department of Quantitative and Computational Biology, Department of Chemistry, Dornsife Center for New Technologies in Drug Discovery and Development, Bridge Institute, Michelson Center for Convergent Bioscience, University of Southern California, Los Angeles, CA 90089, USA

^6^Department of Pharmacology, University of North Carolina School of Medicine, Chapel Hill, NC 27599, USA

^7^Division of Chemical Biology and Medicinal Chemistry, Eshelman School of Pharmacy, University of North Carolina, Chapel Hill, NC, USA

^8^Department of Biochemistry and Molecular Biology, University of Maryland Baltimore, Baltimore, MD, USA.

^9^Washington University Pain Center, Washington University in St. Louis, St. Louis, MO, USA.

^#^Present address: Department of Pharmacological Sciences, Department of Neuroscience, Icahn School of Medicine at Mount Sinai, New York, NY, USA

Corresponding authors: Jon Fay (jfay@som.umaryland.edu)

Tao Che (taoche@wustl.edu)

| **Table of contents** | **Page** |
| --- | --- |
| Section1. Supplementary Methods | 3 |
| Section2. Supplementary Figures | 4 |
| Section3. Supplementary Tables | 10 |

**Supplementary Methods**

**Allosteric IC50 shift model equation**

To determine the binding affinities (KB) between KOR and four G proteins, as well as the ternary complex constant alpha (α), data (with or without normalization) were analyzed with GraphPad Prism 9.3.1 using an allosteric IC50 shift model.

$$Y=Bottom+(Top-Bottom)/(1+{10}^{(LogIC50*\frac{1+\frac{C}{KB}}{1+*\frac{C}{KB}}-X)*Hillslope})$$

X is the log(concentration) of agonist

C is the concentration (not log) of modulator, entered in column titles

IC50 is the concentration of agonist that inhibits half maximal response in the absence of modulator.

KB is the equilibrium dissociation constant (Molar) of modulator binding to its allosteric site.

α is the ternary complex constant. When alpha=1.0, the modulator won't alter binding. If alpha is less than 1.0, then the modulator reduces ligand binding. If alpha is greater than 1.0, then the modulator increases binding.

Top and Bottom are plateaus in the units of the Y axis.

**Supplementary Figures**

**Supplementary Figure 1. Cryo-EM analysis KOR-G protein complexes bound to momSalB or GR89,696.**

**
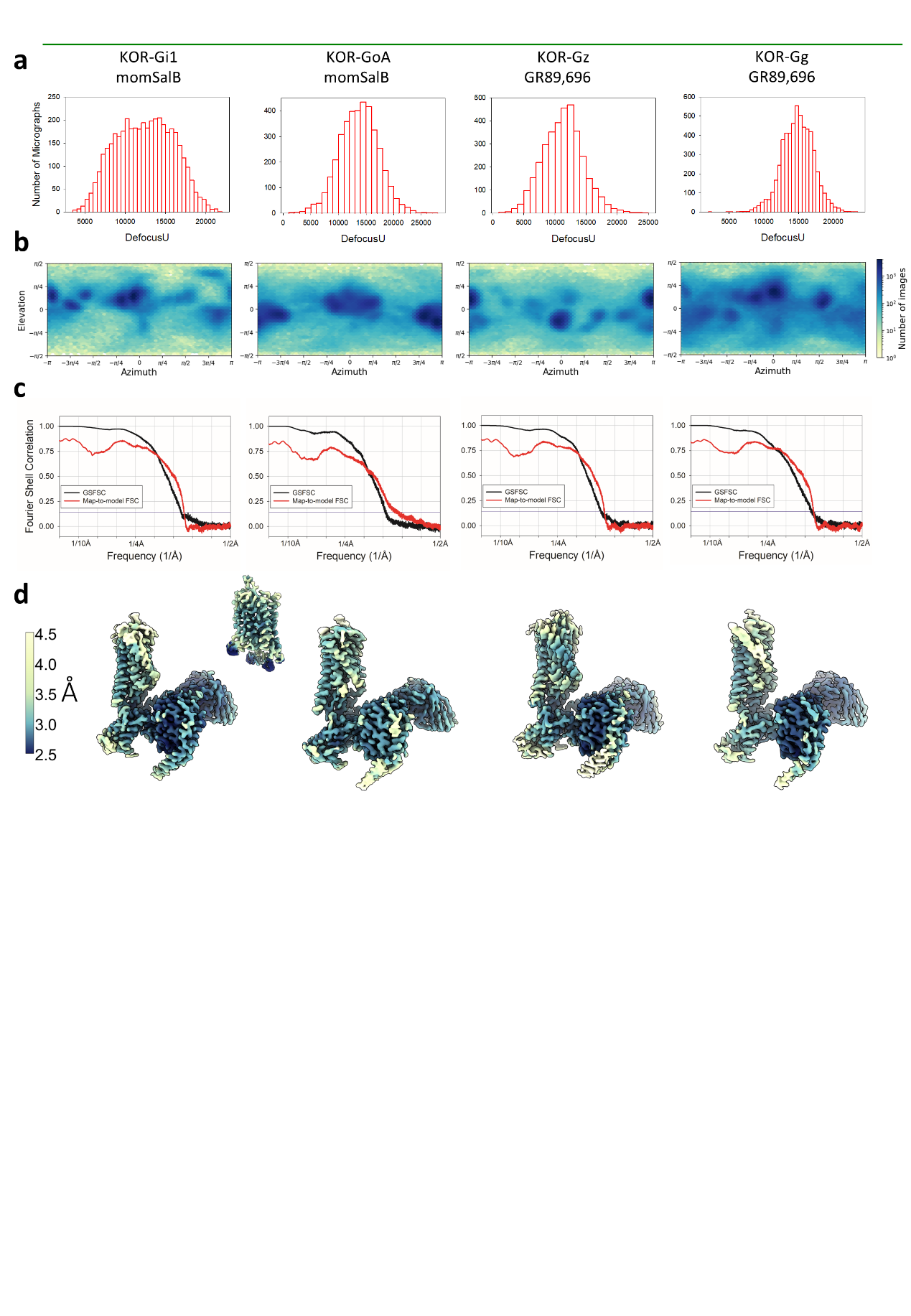
**

Respective agonist-bound KOR heterotrimeric complexes are shown: **a.** Histograms of defocus values for micrographs used in the single-particle analysis (see Extended Data Table 1 for more details). **b.** Orientational distribution heat map. **c.** 2D plots of the gold-standard Fourier shell correlation (GSFSC) between half maps (black) and FSC between the model and the B-factor sharpened map for respective refined model (red) as calculated by Phenix_mtriage. **d.** Local resolution heat-map calculated using the local windowed FSC method. For each complex map, a local refined map for receptor only was produced to improve the resolution of ligand binding pose and receptor conformation. Here shows the local map of KOR in the KOR-Gi1 complex.

**Supplementary Figure 2. Electron microscopy density maps of KOR-G protein complexes bound to momSalB or GR89,696.**

**
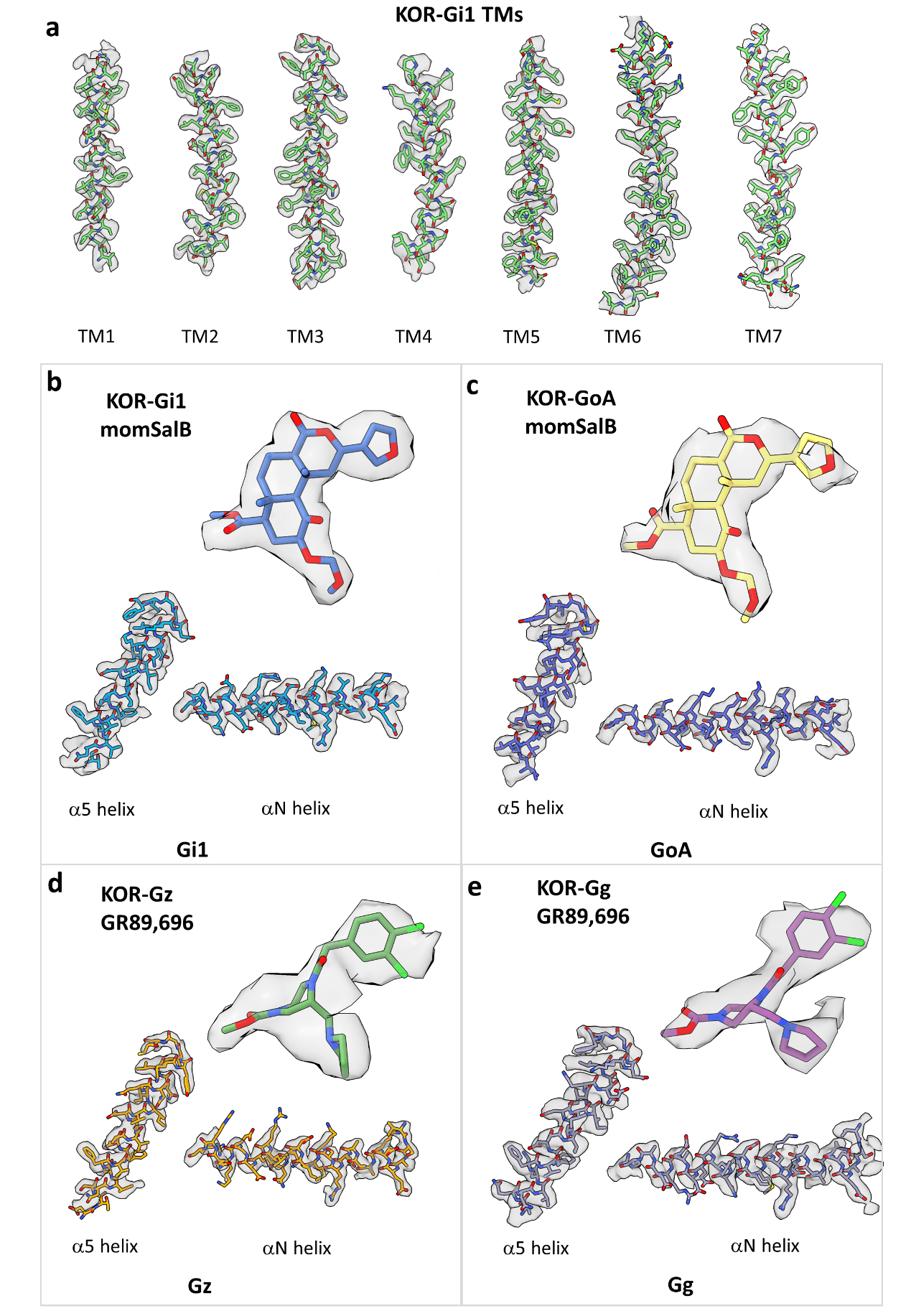
**

**a**. EM density of the TM1-TM7 helices of KOR. **b-e.** EM density of respective agonist and G protein α5 and αN helices.

**Supplementary Figure 3. Comparison of ligand-receptor interactions.**

**
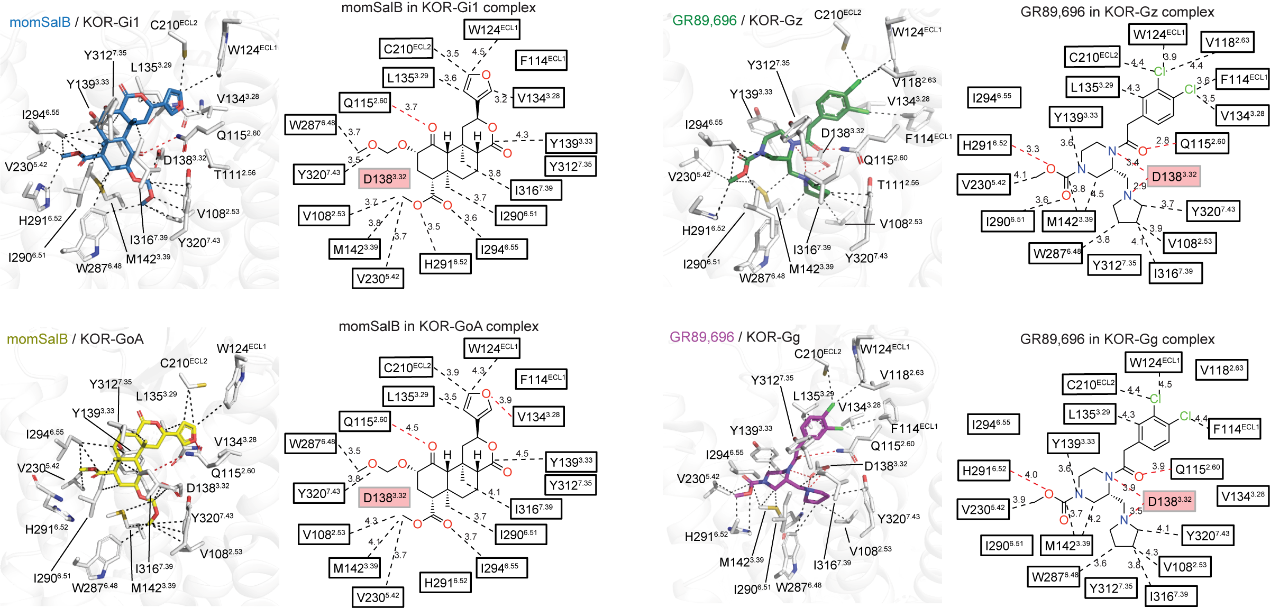
**

The dash line represents the closest distance between the ligand and residues. H-bond or salt-bridge interactions are shown in red. The distance cutoff is 4.5 Å.

**Supplementary Figure 4. The role of intracellular KOR residues on Gi1, GoA, Gz, and Gg coupling, respectively.**

**
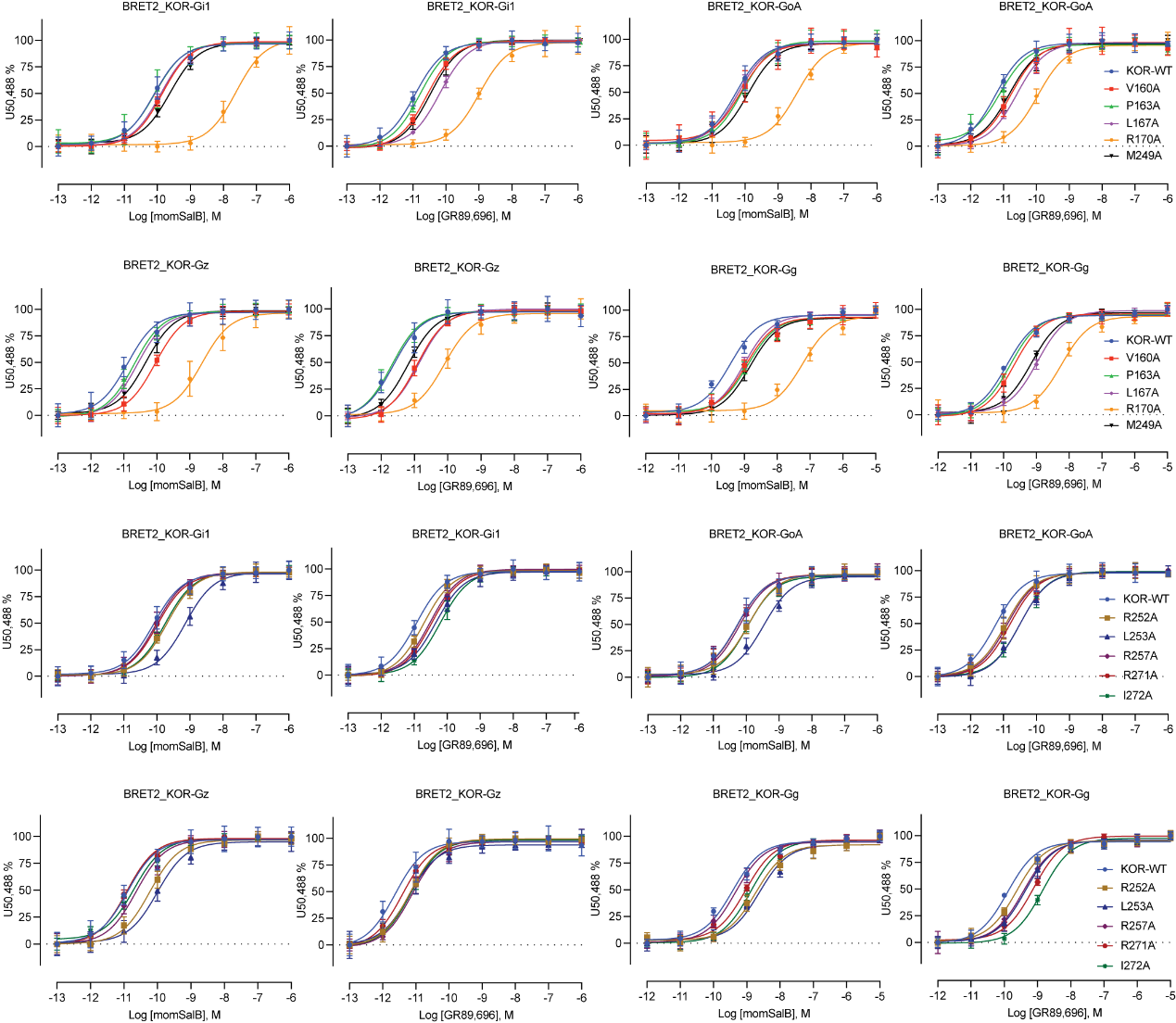
**

The effects of individual KOR mutations on specific Gi1, GoA, Gz, and Gg were screened by the BRET2 KOR-G protein assays. Data are grouped data ± s.e.m. of n = 3 biological replicates. Full quantitative parameters from this experiment are listed in Supplementary Table 16.

**Supplementary Figure 5. Evaluation of different expression levels of Gα proteins.**

**­­­­
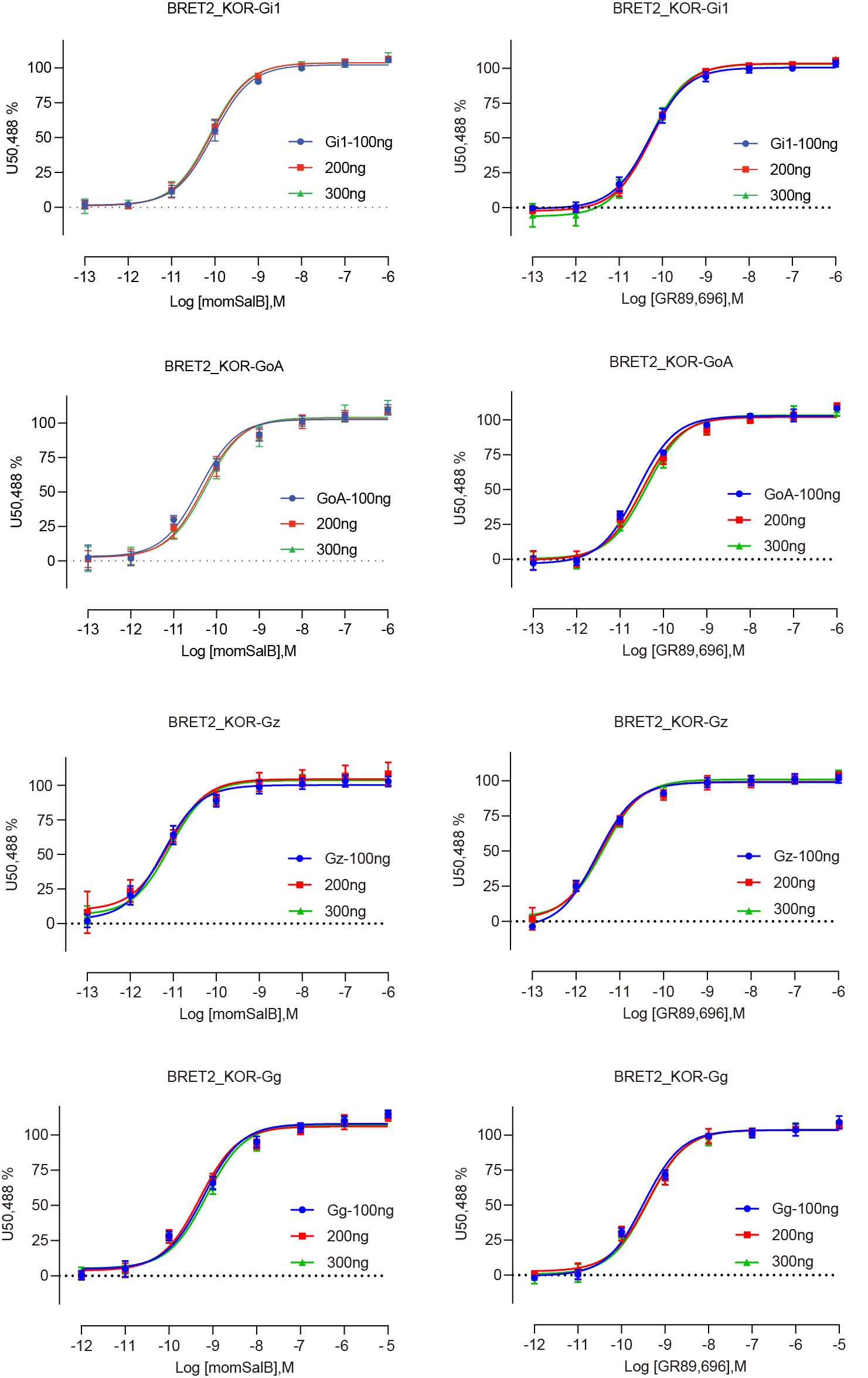
**

Some mutants led to a 1.5 to 1.7-fold changes of G protein expression, and the BRET2 assays confirmed that these altered G proteins have minimal effects on agonist-mediated KOR-G protein coupling. Data are grouped BRET ratio ± s.e.m. from n = 3 biological replicates. Full quantitative parameters from this experiment are listed in Supplementary Table 17.

**Supplementary Figure 6. NMR spectroscopy of synthesized momSalB.**

**
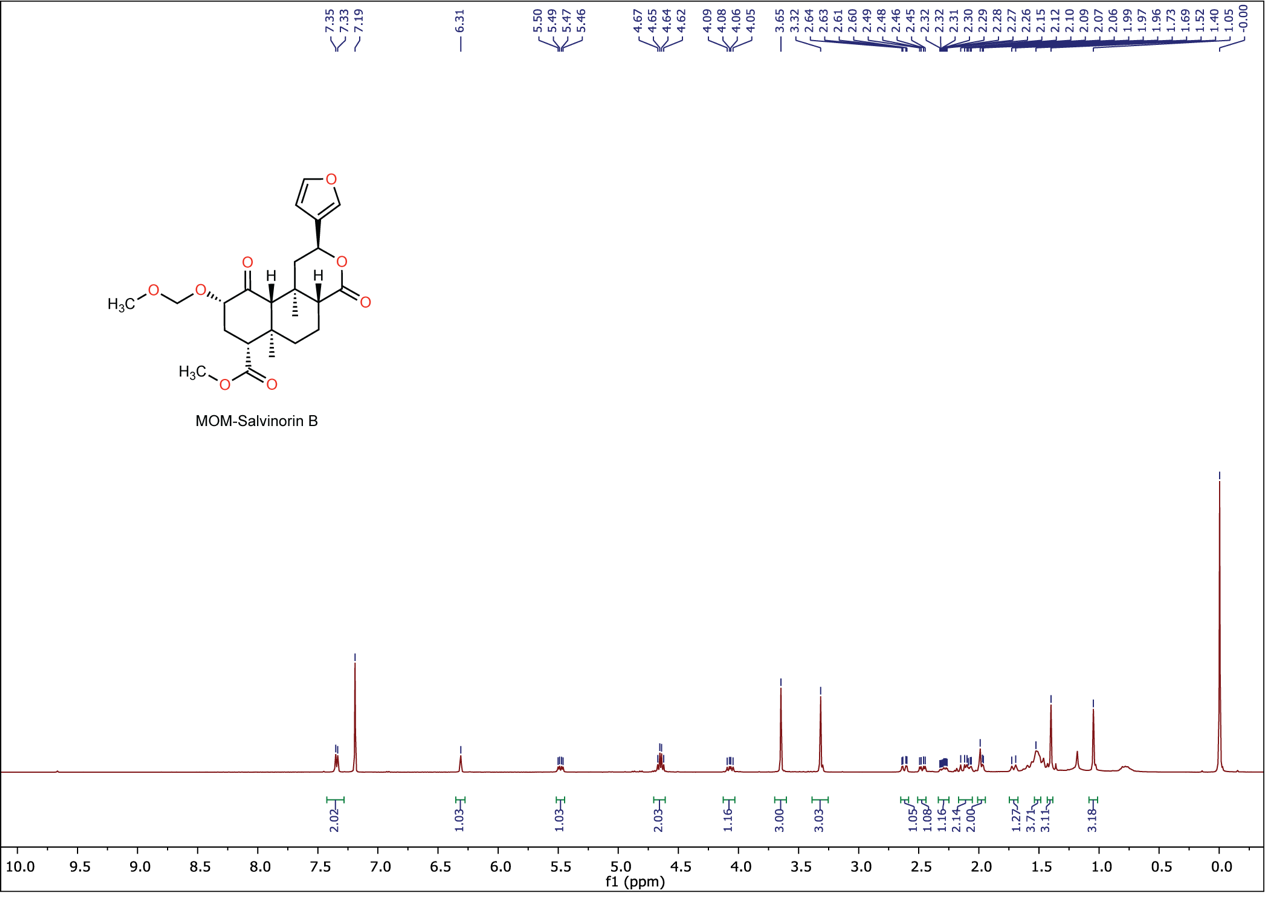
**

momSalB was synthesized via the method described by Lee et al (ref 57). 1H NMR spectra were recorded using tetramethylsilane (TMS) as an internal standard at ambient temperature with a Varian 400 MHz instrument at 400 MHz for 1H NMR spectroscopy. Splitting patterns were designated as singlet (s), broad singlet (br s), doublet (d), triplet (t), quartet (q), quintet (quin), doublet of doublets (dd), and triplet of doublets (td). Splitting patterns that could not be interpreted or easily visualized were designated as multiplets (m).

**Supplementary Tables**

**Supplementary Table 1.** **The role of D138^3.32^N in momSalB, GR89,696 or U50,488-mediated KOR activation**. Data were acquired by cAMP-inhibition assay. Values represent mean pEC50 ± standard error from three independent biological replicates. Significance analyses were performed using unpaired two-tailed student’s t-test to compare each mutant to the wild-type receptor. N.A., no activity (fold-change of EC_50_ > 10^6^), asterisk (****) represents *p*<0.0001.

| Receptor | pEC_50_ ± s.e.m. (cAMP inhibition), M | | |
| --- | --- | --- | --- |
|  | momSalB | GR89,696 | U50,488 |
| KOR WT | 11.36 ± 0.10 | 11.54 ± 0.05 | 9.60 ± 0.10 |
| KOR D138N | 11.28 ± 0.09 | 8.55 ± 0.05**** | N.A.**** |

**Supplementary Table 2.** **Mutagenesis screening of binding pocket residues using G protein-mediated cAMP inhibition assays.** Values represent mean pEC50 ± standard error from three independent biological replicates. Significance analyses were performed using one-way ANOVA. If significant, a Dunnett’s multiple comparisons test was used to compare each mutant to the wild-type receptor. N.A., no activity (fold-change of EC_50_ > 10^7^). Asterisk (*) represents *p*<0.05, asterisk (**) represents *p*<0.01, asterisk (***) represents *p*<0.001, asterisk (****) represents *p*<0.0001.

| Receptor | pEC_50_ ± s.e.m. (cAMP inhibition), M | |
| --- | --- | --- |
|  | momSalB | GR89,696 |
| KOR WT | 11.22 ± 0.04 | 11.79 ± 0.03 |
| KOR V108A | 8.82 ± 0.05**** | 11.37 ± 0.05** |
| KOR Q115N | 10.67 ± 0.03** | 11.54 ± 0.04 |
| KOR W124A | N.A.*** | 9.50 ± 0.08**** |
| KOR V134A | 10.56 ± 0.04** | 11.41 ± 0.05 |
| KOR I135A | 9.19 ± 0.04*** | 10.51 ± 0.05**** |
| KOR M142A | 8.55 ± 0.09**** | 9.87 ± 0.06**** |
| KOR V230A | 9.52 ± 0.05**** | 11.25 ± 0.03* |
| KOR H291A | 8.99 ± 0.09**** | 11.01 ± 0.04*** |
| KOR I316A | 7.77 ± 0.04**** | 11.13 ± 0.06 |
| KOR G319L | 7.49 ± 0.08**** | 9.47 ± 0.06**** |
| KOR Y320L | N.A.**** | 8.88 ± 0.05**** |

**Supplementary Table 3. Measurement of momSalB- or GR89,696- mediated G protein subtype activation by BRET2.** Values represents mean pEC50 ± standard error from four independent biological replicates.

| Ligand | pEC_50_ ± s.e.m (BRET2), M | | | |
| --- | --- | --- | --- | --- |
|  | Gi1 | GoA | Gz | Gg |
| momSalB | 10.13 ± 0.05 | 10.12 ± 0.05 | 10.83 ± 0.05 | 9.02 ± 0.05 |
| GR89,696 | 10.86 ± 0.05 | 11.14 ± 0.05 | 11.62 ± 0.05 | 9.59 ± 0.06 |

**Supplementary Table 4.** **Mutagenesis analysis of R156^3.50^ and N336^8.49^ by BRET2.** Values represent mean pEC50 ± standard error from three independent biological replicates. Significance analyses were performed using the unpaired two-tailed student’s t-test to compare each mutant to the wild-type receptor. N.A. represents no activity. Asterisk (*) represents *p*<0.05, asterisk (**) represents *p*<0.01, asterisk (***) represents *p*<0.001, asterisk (****) represents *p*<0.0001.

| Receptor | momSalB, pEC_50_ ± s.e.m. (BRET2), M | | | | GR89,696, pEC_50_ ± s.e.m. (BRET2), M | | | |
| --- | --- | --- | --- | --- | --- | --- | --- | --- |
|  | Gi1 | GoA | Gz | Gg | Gi1 | GoA | Gz | Gg |
| KOR WT | 10.01 ± 0.06 | 10.44 ± 0.06 | 10.67 ± 0.06 | 9.20 ± 0.05 | 10.71 ± 0.06 | 10.94 ± 0.06 | 11.54 ± 0.06 | 9.69 ± 0.05 |
| KOR R156A | 7.80 ± 0.06**** | 7.63 ± 0.07**** | 8.00 ± 0.06**** | 7.09 ± 0.16*** | 8.00 ± 0.06*** | 8.87 ± 0.06*** | 9.15 ± 0.06*** | 6.76 ± 0.15**** |
| KOR WT | 10.09 ± 0.06 | 10.36 ± 0.05 | 10.74 ± 0.05 | 9.40 ± 0.04 | 10.61 ± 0.06 | 10.65 ± 0.05 | 11.37 ± 0.05 | 9.68 ± 0.04 |
| KOR N336A | 9.76 ± 0.06 | 9.23 ± 0.05**** | 9.77 ± 0.05**** | N.A. | 10.30 ± 0.06 | 9.73 ± 0.06*** | 10.12 ± 0.05**** | N.A. |

**Supplementary Table 5. The effects of G protein subtypes on agonist binding at KOR.** Data were acquired by radio ligand saturation binding assay. Values represent mean Bmax ± standard error from four independent biological replicates. Significance analyses were performed using the unpaired two-tailed student’s t-test to compare groups of KOR stabilized with a heterotrimeric G protein to KOR only. Asterisk (*) represents *p*<0.05, asterisk (**) represents *p*<0.01, asterisk (***) represents *p*<0.001, asterisk (****) represents *p*<0.0001.

| Receptor | Saturation binding ^3^H-U69,593 | | | | |
| --- | --- | --- | --- | --- | --- |
|  | KOR only | KOR + Gi1 | KOR + GoA | KOR + Gz | KOR + Gg |
| Bmax, fmol/mg | 1350 ± 116 | 84324 ± 4214*** | 52086 ± 2465**** | 18623 ± 1468** | 9866 ± 3493** |

**Supplementary Table 6. The allosteric differences between G protein subtypes.** Data were acquired by radio ligand competitive binding assay. Values represent mean K_B_ or Logα ± standard error from three independent biological replicates. Significance analyses were performed using the unpaired two-tailed student’s t-test to compare KB and Logα between G proteins. Asterisk (*) represents *p*<0.05, asterisk (**) represents *p*<0.01, asterisk (***) represents *p*<0.001, asterisk (****) represents *p*<0.0001.

|  | GR89,696, K_B_ ± s.e.m. (radioligand binding), nM | | | |
| --- | --- | --- | --- | --- |
|  | Gi1 | GoA | Gz | Gg |
| K_B_ | 341.0 ± 10.82 | 560.3 ± 19.84 | 1076. ± 59.82 | 1551 ± 16.56 |
|  | GR89,696, Logα ± s.e.m. (radioligand binding), nM | | | |
| Logα | 1.44 ± 0.05 | 1.19 ± 0.06 | 0.95 ± 0.07 | 0.85 ± 0.12 |

|  | Unpaired two-tailed student’s t-test | |
| --- | --- | --- |
|  | KB | Logα |
| Gi1 vs GoA | ****P*=0.0006 | ***p*=0.0014 |
| Gi1 vs Gz | ****p*=0.0003 | ****p*=0.0007 |
| Gi1 vs Gg | *****p*<0.0001 | ****p*=0.0002 |
| GoA vs Gz | ***p*=0.0012 | **p*=0.0432 |
| GoA vs Gg | *****p*<0.0001 | ***p*=0.0037 |
| Gg vs Gz | ***p=*0.0016 | **p*=0.0485 |

**Supplementary Table 7. The effect of GDP or GTP on the allosteric activity of Gi1.** Data were acquired by radio ligand saturation binding assay. Values represent mean Bmax ± standard error from three independent biological replicates. Significance analyses were performed using the unpaired two-tailed student’s t-test to compare groups with or without GDP/GTP. Asterisk (*) represents *p*<0.05, asterisk (**) represents *p*<0.01, asterisk (***) represents *p*<0.001, asterisk (****) represents *p*<0.0001

| Receptor | Saturation binding ^3^H-U69,593 | | |
| --- | --- | --- | --- |
|  | KOR only | KOR + GDP | KOR + GTP |
| Bmax, fmol/mg | 5094 ± 403 | 5109 ± 305 | 5566 ± 394 |

| Gi1 | Saturation binding ^3^H-U69,593 | | |
| --- | --- | --- | --- |
|  | KOR + Gi1 | KOR + Gi1 + GDP | KOR + Gi1 + GTP |
| Bmax, fmol/mg | 68030 ± 2335 | 19783 ± 1413**** | 21596 ± 428**** |

**Supplementary Table 8. The allosteric effects of Gi1 and Nb39 on the binding affinity of KOR agonists.** Values represent mean pKi ± standard error from three independent biological replicates.

| Ligand | pK_i_ ± s.e.m (radioligand binding), M | | |
| --- | --- | --- | --- |
|  | KOR | KOR + Gi1 | KOR + Nb39 |
| Dyn A, 1-17 | 7.46 ± 0.04 | 8.45 ± 0.04 | 9.24 ± 0.03 |
| momSalB | 7.50 ± 0.04 | 8.37 ± 0.03 | 9.21 ± 0.04 |

**Supplementary Table 9. The effect of different KOR expression levels on the potency of agonist-mediated cAMP inhibition.** Values represent mean pEC50 ± standard error from three independent biological replicates.

| Receptor | pEC_50_ ± s.e.m (cAMP inhibition), M | |
| --- | --- | --- |
|  | momSalB | GR89,696 |
| KORwt-200ng | 11.04 ± 0.06 | 11.63 ± 0.08 |
| KORwt-400ng | 11.39 ± 0.05 | 11.72 ± 0.06 |
| KORwt-600ng | 11.14 ± 0.06 | 11.24 ± 0.08 |

**Supplementary Table 10. The effects of additional D138^3.32^N mutation on the momSalB, GR89,696, and U50,488-mediated cAMP inhibition through KOR.** Values represent mean pEC50 ± standard error from three independent biological replicates. Significance analyses were performed using the unpaired two-tailed student’s t-test to compare each mutant to the wild-type receptor or double mutation to the single mutation. N.A. represents no activity. Asterisk (*) represents *p*<0.05, asterisk (**) represents *p*<0.01, asterisk (***) represents *p*<0.001, asterisk (****) represents *p*<0.0001. ‘ns’ represents no significance.

| Receptor | pEC_50_ ± s.e.m. (cAMP inhibition), M | | |
| --- | --- | --- | --- |
|  | U50,488 | GR89,696 | momSalB |
| KOR-WT | 9.44 ± 0.07 | 11.78 ± 0.05 | 11.41 ± 0.04 |
| KOR-Q115N | 9.94 ± 0.06 | 11.37 ± 0.04 | 10.48 ± 0.05 |
| KOR-Q115N-D138N | N.A. | 8.88 ± 0.04 | 10.78 ± 0.03 |
| KOR WT | 9.42 ± 0.10 | 11.75 ± 0.06 | 11.41 ± 0.04 |
| KOR-M142A | 7.24 ± 0.12 | 10.06 ± 0.06 | 8.66 ± 0.06 |
| KOR-M142A-D138N | N.A. | N.A. | 8.65 ± 0.07 |
| KOR WT | 9.64 ± 0.08 | 11.96 ± 0.04 | 11.52 ± 0.05 |
| KOR-V230A | 9.01± 0.07 | 11.38 ± 0.05 | 9.49 ± 0.05 |
| KOR-V230A-D138N | N.A. | 7.42 ± 0.06 | 9.89 ± 0.05 |
| KOR WT | 9.51 ± 0.06 | 11.81 ± 0.05 | 11.55 ± 0.06 |
| KOR-H291A | 9.25 ± 0.06 | 10.48 ± 0.06 | 9.08 ± 0.06 |
| KOR-H291A-D138N | N.A. | 7.38 ± 0.06 | 9.95 ± 0.06 |

| Receptor | Unpaired two-tailed student’s t-test | | |
| --- | --- | --- | --- |
|  | U50,488 | GR89,696 | momSalB |
| KOR-WT vs KOR-Q115N | **p*=0.0189 | ***p*=0.0090 | *****p*<0.0001 |
| KOR-WT vs KOR-Q115N-D138N |  | *****p*<0.0001 | ***p*=0.0033 |
| KOR-Q115N vs KOR-Q115N-D138N |  | *****p*<0.0001 | **p*=0.0426 |
| KOR WT vs KOR-M142A | *****p*<0.0001 | *****p*<0.0001 | *****p*<0.0001 |
| KOR WT vs KOR-M142A-D138N |  |  | *****p*<0.0001 |
| KOR-M142A vs KOR-M142A-D138N |  |  | (ns) *p*=0.7625 |
| KOR WT vs KOR-V230A | **p*=0.0137 | ***p*=0.0076 | *****p*<0.0001 |
| KOR WT vs KOR-V230A-D138N |  | *****p*<0.0001 | *****p*<0.0001 |
| KOR-V230A vs KOR-V230A-D138N |  | *****p*<0.0001 | ***p*=0.0034 |
| KOR WT vs KOR-H291A | (ns) *p*=0.0575 | ****p*=0.0005 | *****p*<0.0001 |
| KOR WT vs KOR-H291A-D138N |  | *****p*<0.0001 | *****p*<0.0001 |
| KOR-H291A vs KOR-H291A-D138N |  | *****p*<0.0001 | ***p*=0.0035 |

**Supplementary Table 11. The effects of mutations in the hydrophobic pocket on the binding affinity of momSalB.** Data were acquired by radio ligand competition binding assay. Values represent mean pKi ± standard error from three independent biological replicates.

| Receptor | momSalB, pK_i_ ± s.e.m (radioligand binding), M |
| --- | --- |
| KOR WT | 9.11 ± 0.06 |
| KOR V108A | 8.82 ± 0.05 |
| KOR G319L | 8.53 ± 0.06 |
| KOR Y320L | 8.56 ± 0.06 |

**Supplementary Table 12.** **Mutagenesis analysis of intracellular KOR residues by the G protein-mediated cAMP inhibition assays.** Values represent mean pEC50 ± standard error from three independent biological replicates. Significance analyses were performed using one-way ANOVA. If significant, a Dunnett’s multiple comparisons test was used to compare each mutant to the wild-type receptor. Asterisk (*) represents *p*<0.05, asterisk (**) represents *p*<0.01, asterisk (***) represents *p*<0.001, asterisk (****) represents *p*<0.0001.

| Receptor | pEC_50_ ± s.e.m. (cAMP inhibition), M | |
| --- | --- | --- |
|  | momSalB | GR89,696 |
| KOR WT | 11.11 ± 0.04 | 11.67 ± 0.03 |
| KOR R156A | 7.92 ± 0.05**** | 8.78 ± 0.04**** |
| KOR V160A | 10.46 ± 0.04**** | 10.95 ± 0.03*** |
| KOR P163A | 9.97 ± 0.05**** | 11.12 ± 0.04** |
| KOR L167A | 10.37 ± 0.04*** | 10.63 ± 0.04**** |
| KOR R170A | 8.66 ± 0.05**** | 9.75 ± 0.04**** |
| KOR M249A | 9.56 ± 0.04**** | 10.63 ± 0.04**** |
| KOR R252A | 9.84 ± 0.05**** | 11.08 ± 0.04** |
| KOR L253A | 10.20 ± 0.06**** | 11.07± 0.05*** |
| KOR R257A | 11.10 ± 0.05 | 11.03 ± 0.04*** |
| KOR R271A | 10.63 ± 0.06*** | 11.06 ± 0.05*** |
| KOR I272A | 10.26 ± 0.05*** | 10.63 ± 0.04**** |
| KOR N336A | 9.91 ± 0.05**** | 9.86 ± 0.04**** |

**Supplementary Table 13. The effects of different receptor concentration on KOR mediated Gi1 activation.** Values represent mean pEC50 ± standard error from three independent biological replicates. Significance analyses were performed using one-way ANOVA**.**

|  | pEC_50_ ± s.e.m (BRET2), M | |
| --- | --- | --- |
|  | Gαi1 | |
| Ligand | momSalB | GR89,696 |
| KORwt (100 ng) | 9.89± 0.07 | 10.90 ± 0.06 |
| KORwt (200 ng) | 9.97 ± 0.06 | 10.83 ± 0.04 |
| KORwt (300 ng) | 10.12 ± 0.03 | 10.77 ± 0.03 |
| KORwt (400 ng) | 10.13 ± 0.03 | 10.85 ± 0.02 |
| KORwt (500 ng) | 10.17 ± 0.03 | 10.81 ± 0.03 |
| KORwt (600 ng) | 10.20 ± 0.03 | 10.81 ± 0.04 |

**Supplementary Table 14. The roles of nonconserved residues of each G protein-α5 helix on the KOR-G protein interaction.** Values represent mean pEC50 ± standard error from three independent biological replicates. Significance analyses were performed using one-way ANOVA. If significant, a Dunnett’s multiple comparisons test was used to compare each mutant to the wild-type G protein. Asterisk (*) represents *p*<0.05, asterisk (**) represents *p*<0.01, asterisk (***) represents *p*<0.001, asterisk (****) represents *p*<0.0001.

| Gi1 | pEC_50_ ± s.e.m. (BRET2), M | |
| --- | --- | --- |
|  | momSalB | GR89,696 |
| WT | 10.11 ± 0.06 | 11.04 ± 0.04 |
| K345A | 10.21 ± 0.06 | 11.03 ± 0.04 |
| D350A | 10.12 ± 0.06 | 10.96 ± 0.04 |
| C351A | 9.59 ± 0.06 | 10.44 ± 0.03*** |
| F354A | 9.84 ± 0.05 | 10.57 ± 0.03 |

| GoA | pEC_50_ ± s.e.m. (BRET2), M | |
| --- | --- | --- |
|  | momSalB | GR89,696 |
| WT | 10.19 ± 0.03 | 11.10 ± 0.04 |
| A345Q | 10.16 ± 0.04 | 10.95 ± 0.04 |
| R349A | 9.88 ± 0.03* | 10.50 ± 0.03** |
| G350A | 10.18 ± 0.05 | 11.03 ± 0.05 |
| C351A | 9.96 ± 0.04* | 10.73 ± 0.04* |
| Y354A | 10.15 ± 0.04 | 10.88 ± 0.04 |

| Gz | pEC_50_ ± s.e.m. (BRET2 assay), M | |
| --- | --- | --- |
|  | momSalB | GR89,696 |
| WT | 10.87 ± 0.05 | 11.73 ± 0.05 |
| R31A | 10.82 ± 0.03 | 11.52 ± 0.03 |
| Q346A | 11.18 ± 0.05 | 11.92 ± 0.03 |
| Y351A | 10.97 ± 0.07 | 11.75 ± 0.08 |
| I352A | 9.98 ± 0.05*** | 10.91 ± 0.04**** |
| C355A | 10.65 ± 0.06 | 11.49 ± 0.05 |

| Gg | pEC_50_ ± s.e.m. (BRET2 assay), M | |
| --- | --- | --- |
|  | momSalB | GR89,696 |
| WT | 9.34 ± 0.06 | 9.72 ± 0.07 |
| K345A | 9.28 ± 0.07 | 10.06 ± 0.07 |
| E346A | 9.55 ± 0.07 | 10.01 ± 0.06 |
| D350A | 9.54 ± 0.07 | 10.00 ±0.09 |
| C351A | 9.12 ± 0.10* | 9.78 ± 0.10 |
| F354A | 9.42 ± 0.08 | 9.87 ± 0.09 |

**Supplementary Table 15. The effect of GDP or GTP on the allosteric activity of GoA, Gz, and Gg.** Data were acquired by radio ligand saturation binding assay. Values represent mean Bmax ± standard error from three independent biological replicates. Significance analyses were performed using the unpaired two-tailed student’s t-test to compare groups with or without GDP or GTP. (*) represents p values compared to KOR + GoA, KOR + Gz, or KOR + Gg, respectively. Asterisk (*) represents *p*<0.05, asterisk (**) represents *p*<0.01, asterisk (***) represents *p*<0.001, asterisk (****) represents *p*<0.0001.

| GoA | Saturation binding ^3^H-U69,593 | | |
| --- | --- | --- | --- |
|  | KOR + GoA | KOR + GoA + GDP | KOR + GoA + GTP |
| Bmax, fmol/mg | 8890 ± 239 | 3081 ± 196**** | 6241 ± 689*** |

| Gz | Saturation binding ^3^H-U69,593 | | |
| --- | --- | --- | --- |
|  | KOR + Gz | KOR + Gz + GDP | KOR + Gz + GTP |
| Bmax, fmol/mg | 3446 ± 220 | 942 ± 82**** | 890 ± 47**** |

| Gg | Saturation binding ^3^H-U69,593 | | |
| --- | --- | --- | --- |
|  | KOR + Gg | KOR + Gg + GDP | KOR + Gg + GTP |
| Bmax, fmol/mg | 1592 ± 141 | 645 ± 186*** | 771 ± 46*** |

**Supplementary Table 16. The role of intracellular KOR residues on Gi1, GoA, Gz and Gg coupling.** Data were acquired by BRET2 assay. Values represent mean pEC50 ± standard error from three independent biological replicates. Significance analyses were performed using one-way ANOVA. If significant, a Dunnett’s multiple comparisons test was used to compare each mutant to the wild-type receptor. Asterisk (*) represents *p*<0.05, asterisk (**) represents *p*<0.01, asterisk (***) represents *p*<0.001, asterisk (****) represents *p*<0.0001.

| KOR mutation | pEC_50_ ± s.e.m. (BRET2 assay), M | | | | | | | |
| --- | --- | --- | --- | --- | --- | --- | --- | --- |
|  | momSalB | | | | GR89,696 | | | |
|  | Gi1 | GoA | Gz | Gg | Gi1 | GoA | Gz | Gg |
| KOR WT | 10.10 ± 0.06 | 10.28 ± 0.07 | 10.89 ± 0.07 | 9.42 ± 0.05 | 10.93 ± 0.05 | 11.24 ± 0.06 | 11.63 ± 0.08 | 9.90 ± 0.04 |
| V160A | 9.86 ± 0.03 | 10.13 ± 0.10 | 10.04 ± 0.04**** | 8.96 ± 0.06** | 10.56 ± 0.03** | 10.76 ± 0.08 | 10.93 ± 0.05**** | 9.68 ± 0.05 |
| P163A | 10.07 ± 0.07 | 10.09 ± 0.07 | 10.68 ± 0.05 | 8.89 ± 0.06**** | 10.79 ± 0.03 | 11.10 ± 0.06 | 11.68 ± 0.06 | 9.80 ± 0.04 |
| L167A | 9.90 ± 0.02 | 10.22 ± 0.04 | 10.60 ± 0.03 | 9.01 ± 0.04** | 10.18 ± 0.02**** | 10.59 ± 0.04** | 10.86 ± 0.02**** | 8.97 ± 0.03**** |
| R170A | 7.61 ± 0.06**** | 8.42 ± 0.06**** | 8.63 ± 0.09**** | 7.22 ± 0.07**** | 8.99 ± 0.05**** | 9.96 ± 0.05**** | 10.10 ± 0.06**** | 8.23 ± 0.06**** |
| M249A | 9.61 ± 0.04*** | 9.91 ± 0.05** | 10.33 ± 0.04** | 8.83 ± 0.06**** | 10.47 ± 0.03*** | 10.83 ± 0.05 | 11.19 ± 0.04** | 9.14 ± 0.05**** |
| R252A | 9.70 ± 0.04** | 9.93 ± 0.06* | 10.23 ± 0.04*** | 8.73 ± 0.06**** | 10.71 ± 0.04 | 10.94 ± 0.06 | 11.17 ± 0.04** | 9.63 ± 0.04 |
| L253A | 9.14 ± 0.05**** | 9.47 ± 0.05**** | 10.00 ± 0.06**** | 8.62 ± 0.06**** | 10.35 ± 0.04**** | 10.51 ± 0.05**** | 11.19 ± 0.06*** | 9.37 ± 0.05** |
| R257A | 10.03 ± 0.04 | 10.21 ± 0.06 | 10.63 ± 0.06 | 9.34 ± 0.05 | 10.51± 0.04*** | 10.89 ± 0.04 | 11.12 ± 0.05**** | 9.43 ± 0.05** |
| R271A | 9.99 ± 0.03 | 10.26 ± 0.05 | 10.92 ± 0.03 | 9.03 ± 0.04** | 10.47 ± 0.02*** | 10.81 ± 0.05 | 11.40 ± 0.03 | 9.12 ± 0.03**** |
| I272A | 9.75 ± 0.03* | 9.97 ± 0.04* | 10.70 ± 0.05 | 8.88 ± 0.04**** | 10.21 ± 0.04**** | 10.49 ± 0.04*** | 11.15 ± 0.04*** | 8.85 ± 0.03**** |

**Supplementary Table 17. The role of different G protein expression levels on momSalB-mediated KOR activation.** Values represent mean pEC50 ± standard error from three independent biological replicates. Significance analyses were performed using one-way ANOVA. If significant, a Tukey multiple comparisons test was used to compare one G protein at three transfection concentrations. Asterisk (*) represents *p*<0.05, asterisk (**) represents *p*<0.01, asterisk (***) represents *p*<0.001, asterisk (****) represents *p*<0.0001.

|  | pEC_50_ ± s.e.m. (BRET2), M | | |
| --- | --- | --- | --- |
| Ligand | momSalB | | |
| G protein | 100 ng | 200 ng | 300 ng |
| Gi1 | 10.03± 0.03 | 10.08 ± 0.03 | 10.10 ± 0.03 |
| GoA | 10.40 ± 0.06 | 10.31 ± 0.05 | 10.25 ± 0.06 |
| Gz | 11.23 ± 0.05 | 11.10 ± 0.07 | 11.08 ± 0.04 |
| Gg | 9.21 ± 0.05 | 9.30 ± 0.05 | 9.15 ± 0.05 |
