# Supplementary material for: Ligand and G-protein selectivity in the κ-opioid receptor
Source: Nature. 2023 May 3;617(7960):417–25. doi: 10.1038/s41586-023-06030-7 (PMC10172140; doi:10.1038/s41586-023-06030-7)
Supplement: Supplementary file 2 — Reporting Summary [file 41586_2023_6030_MOESM2_ESM.pdf]

nature research

Corresponding author(s): Jonathan F. Fay, Tao Che

Last updated by author(s): Mar 1, 2023

Reporting Summary

Nature Research wishes to improve the reproducibility of the work that we publish. This form provides structure for consistency and transparency in reporting. For further information on Nature Research policies, see Authors & Referees and the Editorial Policy Checklist.

Statistics

For all statistical analyses, confirm that the following items are present in the figure legend, table legend, main text, or Methods section.

n/a	Confirmed	
☐	☒	The exact sample size (*n*) for each experimental group/condition, given as a discrete number and unit of measurement
☐	☒	A statement on whether measurements were taken from distinct samples or whether the same sample was measured repeatedly
☐	☒	The statistical test(s) used AND whether they are one- or two-sided<br>*Only common tests should be described solely by name; describe more complex techniques in the Methods section.*
☒	☐	A description of all covariates tested
☒	☐	A description of any assumptions or corrections, such as tests of normality and adjustment for multiple comparisons
☐	☒	A full description of the statistical parameters including central tendency (e.g. means) or other basic estimates (e.g. regression coefficient) AND variation (e.g. standard deviation) or associated estimates of uncertainty (e.g. confidence intervals)
☐	☒	For null hypothesis testing, the test statistic (e.g. *F*, *t*, *r*) with confidence intervals, effect sizes, degrees of freedom and *P* value noted<br>*Give P values as exact values whenever suitable.*
☒	☐	For Bayesian analysis, information on the choice of priors and Markov chain Monte Carlo settings
☒	☐	For hierarchical and complex designs, identification of the appropriate level for tests and full reporting of outcomes
☒	☐	Estimates of effect sizes (e.g. Cohen's *d*, Pearson's *r*), indicating how they were calculated

Our web collection on statistics for biologists contains articles on many of the points above.

Software and code

Policy information about availability of computer code

Data collection	SerialEM V3.8.2
Data analysis	Structure determination and refinement: cryoSPARC V3.1.0, Topaz 0.2.2, DeepEMhancer, UCSF Chimera 1.15, UCSF ChimeraX 1.3, PyMol 2.4.1, Coot 0.9.5, MolProbity (built in phenix 1.18.2), Phenix 1.18.2<br>Functional data analysis: GraphPad Prism 9<br>Ligand illustrator: ChemDraw 20.0.<br>Docking and molecular simulation: CHARMM-GUI (https://www.charmm-gui.org/), AMBER v20.12 (including CUD and PMEMD), Gromacs simulation (version 2020.3), matplotlib v3.4.3, seaborn v0.11.2, pytraj v2.0.6

For manuscripts utilizing custom algorithms or software that are central to the research but not yet described in published literature, software must be made available to editors/reviewers. We strongly encourage code deposition in a community repository (e.g. GitHub). See the Nature Research guidelines for submitting code & software for further information.

Data

Policy information about availability of data

All manuscripts must include a data availability statement. This statement should provide the following information, where applicable:

- Accession codes, unique identifiers, or web links for publicly available datasets
- A list of figures that have associated raw data
- A description of any restrictions on data availability

The coordinate and cryoEM map of KOR-Gi1-momSalB, KOR-GoA-momSalB, KOR-Gz-GR89,696, and KOR-Gg-GR89,696 have been deposited to PDB and EMDB databases with accession code 8DZP (EMD-27804), 8DZQ (EMD-27805), 8DZS (EMD-27807) and 8DZR (EMD-27806), respectively. PDB database (https://www.rcsb.org/) was used in this study to download the structures of 5-HT2AR-Gq (PDB ID 6WHA), MOR-Gi (PDB ID 6DDE), β2AR-Gs (PDB ID 3SN6) and 5HT1B-miniGo complex (6G79) for structural analysis.

Field-specific reporting

Please select the one below that is the best fit for your research. If you are not sure, read the appropriate sections before making your selection.

☒ Life sciences ☐ Behavioural & social sciences ☐ Ecological, evolutionary & environmental sciences

For a reference copy of the document with all sections, see nature.com/documents/nr-reporting-summary-flat.pdf

Life sciences study design

All studies must disclose on these points even when the disclosure is negative.

Sample size	Sample size was not predetermined by statistical methods. For functional assays (such as cAMP inhibition, BRET2, Radio ligand binding assay, ELISA), there are at least three technical replicates and biological replicates that are reported in the figure legends.<br>For the cryo-EM studies, the number of images is determined by the available microscope time and the requirement of the resolution and 3D reconstruction of EM map. The number of images used for structural determination is sufficient to gain high-resolution maps and build accurate atomic models.
Data exclusions	No data were excluded for this study.
Replication	For functional assay, data were replicated using technical and independent biological replicates. See figure legends for specific details. For the GPCRome assay, one biologically independent experiment was performed with n=4 technical replicates. The four replicates were reliably reproduced.
Randomization	No data is required randomization, because this study did not allocate experimental groups. For cryoEM study, meshes on the grids with good ice thickness were randomly selected for data collection.
Blinding	No blinding was performed in this study. For both cryoEM structure determination and functional studies, blinding is not necessary due to the nature of these experiments do not requires subject assessment of the data that may influence the validity of the results

Reporting for specific materials, systems and methods

We require information from authors about some types of materials, experimental systems and methods used in many studies. Here, indicate whether each material, system or method listed is relevant to your study. If you are not sure if a list item applies to your research, read the appropriate section before selecting a response.

Materials & experimental systems

n/a	Involved in the study
☐	☒ Antibodies
☐	☒ Eukaryotic cell lines
☒	☐ Palaeontology
☒	☐ Animals and other organisms
☒	☐ Human research participants
☒	☐ Clinical data

Methods

n/a	Involved in the study
☒	☐ ChIP-seq
☒	☐ Flow cytometry
☒	☐ MRI-based neuroimaging

Antibodies

Antibodies used	gp64-PE antibody (expression system, #97-201), anti-FLAG-horseradish peroxidase-conjugated antibody (Sigma, A8592)
Validation	gp64-PE antibody was purchased from Expression Systems and was used for baculovirus titration. The detailed information can be found at https://expressionsystems.com/product/gp64-pe-antibody/<br>anti-FLAG-horseradish peroxidase-conjugated antibody was ordered from Sigma-Aldrich and was used in ELISA experiment for the detection of KOR receptor (wt and mutants) expression level. The detailed information can be found at https://www.sigmaaldrich.com/US/en/product/sigma/a8592

Eukaryotic cell lines

Policy information about cell lines

Cell line source(s)	HEK293T cells were purchased from the American Type Culture Collection (ATCC, ATCC CRL-11268). Spodoptera frugiperda (Sf9) cells are from Expression Systems (#94-001S).
Authentication	All cells used in this study are commercial as indicated in the manuscript. HEK293T cells were authenticated by the supplier

Authentication	(ATCC) using morphology and growth characteristics, and STR profiling. Sf9 cells are commercial and obtained from vendors as indicated in the manuscript. No additional authentication was performed by the authors of this study.
Mycoplasma contamination	HEK293T cells have been tested and shown to be free from mycoplasma (Hoechst DNA strain and Direct Culture methods employed). Sf9 cell line was certified as mycoplasma-free by the source company.
Commonly misidentified lines (See ICLAC register)	No commonly misidentified cell lines were used.
